# Rationale and design of the OPTIMIZE trial: OPen label multicenter randomized trial comparing standard IMmunosuppression with tacrolimus and mycophenolate mofetil with a low exposure tacrolimus regimen In combination with everolimus in *de novo* renal transplantation in Elderly patients

**DOI:** 10.1186/s12882-021-02409-8

**Published:** 2021-06-02

**Authors:** S. E. de Boer, J. S.F. Sanders, F. J. Bemelman, M. G.H. Betjes, J. G.M. Burgerhof, L. Hilbrands, D. Kuypers, B. C. van Munster, S. A. Nurmohamed, A. P.J. de Vries, A. D. van Zuilen, D. A. Hesselink, S. P. Berger

**Affiliations:** 1 Department of Internal Medicine, Division of Nephrology, University Medical Center Groningen, University of Groningen, Groningen, The Netherlands; 2 Department of Internal Medicine, Division of Nephrology, Amsterdam Universal Medical Center, Amsterdam, The Netherlands; 3 Department of Internal Medicine, Division of Nephrology & Transplantation, Erasmus MC, Erasmus University Medical Center, Rotterdam, The Netherlands; 4 Department of Epidemiology, University Medical Center Groningen, University of Groningen, Groningen, The Netherlands; 5 Department of Internal Medicine, Division of Nephrology, Radboud University Medical Center, Nijmegen, The Netherlands; 6 Department of Nephrology and Renal Transplantation, University Hospitals Leuven, Leuven, Belgium; 7 Department of Internal Medicine, Divison of Geriatrics, University Medical Center Groningen, University of Groningen, Groningen, The Netherlands; 8 Department of Internal Medicine, Division of Nephrology; and Leiden Transplant Center, Leiden University Medical Center, Leiden University, Leiden, The Netherlands; 9 Department of Internal Medicine, Division of Nephrology, University Medical Center Utrecht, Utrecht, The Netherlands

**Keywords:** Elderly kidney transplant recipients, Reduced CNI exposure, mTOR inhibitor, Everolimus, (Health-related) quality of life, Patient-reported outcomes, Frailty, Immunosenescence, Randomized clinical trial, Multicenter trial

## Abstract

**Background:**

In 2019, more than 30 % of all newly transplanted kidney transplant recipients in The Netherlands were above 65 years of age. Elderly patients are less prone to rejection, and death censored graft loss is less frequent compared to younger recipients. Elderly recipients do have increased rates of malignancy and infection-related mortality. Poor kidney transplant function in elderly recipients may be related to both pre-existing (i.e. donor-derived) kidney damage and increased susceptibility to nephrotoxicity of calcineurin inhibitors (CNIs) in kidneys from older donors. Hence, it is pivotal to shift the focus from prevention of rejection to preservation of graft function and prevention of over-immunosuppression in the elderly. The OPTIMIZE study will test the hypothesis that reduced CNI exposure in combination with everolimus will lead to better kidney transplant function, a reduced incidence of complications and improved health-related quality of life for kidney transplant recipients aged 65 years and older, compared to standard immunosuppression.

**Methods:**

This open label, randomized, multicenter clinical trial will include 374 elderly kidney transplant recipients (≥ 65 years) and consists of two strata. Stratum A includes elderly recipients of a kidney from an elderly deceased donor and stratum B includes elderly recipients of a kidney from a living donor *or* from a deceased donor < 65 years. In each stratum, subjects will be randomized to a standard, tacrolimus-based immunosuppressive regimen with mycophenolate mofetil and glucocorticoids or an adapted immunosuppressive regimen with reduced CNI exposure in combination with everolimus and glucocorticoids. The primary endpoint is ‘successful transplantation’, defined as survival with a functioning graft and an eGFR ≥ 30 ml/min per 1.73 m^2^ in stratum A and ≥ 45 ml/min per 1.73 m^2^ in stratum B, after 2 years, respectively.

**Conclusions:**

The OPTIMIZE study will help to determine the optimal immunosuppressive regimen after kidney transplantation for elderly patients and the cost-effectiveness of this regimen. It will also provide deeper insight into immunosenescence and both subjective and objective outcomes after kidney transplantation in elderly recipients.

**Trial registration:**

ClinicalTrials.gov: NCT03797196, registered January 9th, 2019. EudraCT: 2018-003194-10, registered March 19th, 2019.

**Supplementary Information:**

The online version contains supplementary material available at 10.1186/s12882-021-02409-8.

## Background

### Background and rationale

Elderly patients are an important and growing part of both the dialysis and kidney transplant population. In 2019, 30 % of the newly transplanted kidney transplant recipients in the Netherlands were above 65 years of age, while more than 60 % of the dialysis population was above 65 years of age. These elderly kidney transplant recipients (KTR) have different risk profiles when compared with younger KTR. Whereas graft loss in younger patients is mainly due to loss of the transplant, graft loss in elderly patients is predominantly associated with patient’s death with a functioning graft. Thus, death censored graft loss amongst elderly KTR is a relatively rare phenomenon [1, 2]. There are two important explanations that account for this difference.

First, the incidence of rejection amongst elderly KTR is lower; the aging immune system renders the elderly patient less prone to rejection [1, 3]. This decreased propensity for rejection is explained by senescence of adaptive immunity with decreasing telomere length of immune cells and a shift from a naïve T cell repertoire towards more terminally differentiated memory T cells [4]. Additionally, a shift towards an increasing proportion of CD28^‒^ T cells has been noted [5, 6].

Second, a higher competing incidence of death amongst elderly KTR is observed; mostly because these patients have increased rates of mortality associated with cardiovascular complications, infection, and malignancy [2, 7–11].

Besides recipient characteristics, donor characteristics are also important determinants of outcome in KTR. The Eurotransplant Senior Program (ESP) allocates kidneys within a narrow geographic area from donors aged ≥ 65 years to recipients ≥ 65 years regardless of HLA, thereby minimizing cold ischemia time [12]. Therefore, poor kidney transplant function in these elderly KTR may be related to chronic injury of the kidney prior to transplantation due to older donor age, and to an increased susceptibility of these kidneys to the (nephron-)toxicity of calcineurin inhibitors (CNIs) [13], the cornerstone of current immunosuppressive regimens.

Consequently, it is plausible that elderly KTR might require a specific immunosuppressive approach that balances their decreased propensity for rejection on the one hand, and the increased rates of malignancy, infection-related mortality, and increased CNI susceptibility on the other hand. In these patients the focus needs to be shifted from prevention of rejection to preservation of graft function and prevention of over-immunosuppression. Therefore, we hypothesize that elderly KTR would benefit from reduced CNI exposure.

Currently, complete elimination of CNIs does not seem a realistic option. A Cochrane systematic review showed that the risk ratio for acute rejection is 2.16 with avoidance of CNIs, and 3.21 with late withdrawal [14]. There are no randomized trials that compare immunosuppressive regimens with and without CNIs specifically in the elderly.

The only regimen consisting of registered drugs that successfully may allow avoidance of CNIs is based on the use of CTLA4-Ig, belatacept [15]. However, as belatacept is dependent on blocking the CD80/86 CD28 pathway, this drug may not be ideal in elderly KTR. As described above, immunosenescence is characterized by a loss of CD28 + T cells, and the presence of CD8 + CD28- T cells poses a risk for allograft rejection under belatacept treatment [16]. Literature about the effects of belatacept specifically in the elderly is very sparse. A (small) sub analysis of the elderly (≥ 65 years) recipients in the BENEFIT-EXT study showed a higher incidence (26 %) of acute rejection after 3 years in the low intensity arm of belatacept, versus the cyclosporine arm (14 %) [17]. Additionally, belatacept is administered by in-hospital monthly infusions. This may be an unacceptable burden for a patient population with reduced mobility, and with problematic vascular access.

However, reduction of CNIs in this population seems a realistic option. Previous studies have demonstrated that this reduction is possible when CNIs are combined with an mTOR inhibitor [18–20]. The recent multicenter TRANSFORM trial compared a regimen of everolimus and reduced-exposure CNI, with a regimen of mycophenolate mofetil and standard-exposure CNI in 2037 *de novo* KTR. The study showed that the reduced-exposure CNI regimen offered comparable efficacy and graft function, with low rates of treated biopsy-proven acute rejection and *de novo* donor-specific antibodies, and a significantly lower incidence of viral infections relative to standard-exposure CNI up to two years after transplantation [20].

However, no difference in graft function at two years was present between the standard- and reduced-CNI group. This might be explained by the fact that donors of the included KTR were relatively young, with a mean age of 48.3 years. Hence, these kidneys might have been more resilient towards unfavorable CNI effects. Another explanation could be the smaller difference in CNI exposure between the standard- and reduced-CNI group than intended. The CNI exposure was relatively low in the standard-CNI group and relatively high in the reduced-CNI group. In particular for the reduced-CNI group adherence to the target trough levels as specified in the protocol was suboptimal. The intended concentrations were 4–7 ng/ml during months 0–2, 2–5 ng/ml during months 3–6, and 2–4 ng/ml thereafter [21]. However, the mean concentrations after 1, 4 and 12 months exceeded this target ranges with 7.1, 5.1 and 4.1 ng/ml, respectively. Intended concentrations in the standard-CNI group were 8–12 ng/ml during months 0–2, 6–10 ng/ml during months 3–6, and 5–8 ng/ml thereafter, with actual mean concentrations of 10.3, 8.2 and 6.9 ng/ml, respectively.

### Objectives

The primary objective of the OPTIMIZE study (OPen label multicenter randomized Trial comparing standard IMmunosuppression with tacrolimus and mycophenolate mofetil with a low exposure tacrolimus regimen In combination with everolimus in *de novo* renal transplantation in Elderly patients) is to test the hypothesis that reduced CNI exposure in combination with everolimus will lead to an improved outcome in elderly (≥ 65 years) KTR of A: Kidneys from older deceased donors (≥ 65 years) and B: Kidneys from living donors (all ages) and younger deceased donors (< 65 years). The comparator will be standard CNI exposure in combination with mycophenolate mofetil.

We will also:

- Evaluate the impact of transplantation and adapted immunosuppression on frailty and health-related quality of life in elderly Dutch and Belgian KTR.
- Monitor the function of the aged immune system after transplantation and the effect of low CNI exposure in combination with everolimus on parameters of immunosenescence compared to standard, tacrolimus-based immunosuppression.
- Identify immunologic parameters that may serve as biomarkers of immunosenescence for future clinical application.

## Methods / design

### Design and study population

The OPTIMIZE study is an investigator-driven, randomized, multicenter, open-label, intervention trial. A total of 374 patients will be included. Six Dutch transplant centers (University Medical Center Groningen, Amsterdam Universal Medical Center, Erasmus University Medical Center (Rotterdam), Radboud University Medical Center (Nijmegen), Leiden University Medical Center, and University Medical Center Utrecht) and one Belgian center (UZ Leuven) participate in this study.

The study population consists of *de novo* kidney transplant recipients aged 65 years or older at the time of transplantation. This age limit is primarily chosen because current Eurotransplant allocation includes recipients and donors in ESP from an age of 65 and upward.

The trial will consist of two strata:

- Stratum A: Elderly recipients (≥ 65 years) of kidneys from elderly deceased donors (≥ 65 years) within the Eurotransplant Senior Program.
- Stratum B: Elderly recipients (≥ 65 years) of kidneys from deceased donors (< 65 years) or living donors (all ages).

Most patients in these strata will be eligible for the study. The inclusion and exclusion criteria are shown in Table 1. In summary, patients are only excluded if there is a high or a very low immunological risk, if there are active infections, or if there is a high chance of unacceptable side-effects of the study medication.

**Table 1:** Inclusion and exclusion criteria for the OPTIMIZE study

Inclusion criteria	Exclusion criteria (for both strata)
1. Written informed consent must be obtained before any assessment is performed	1. Subject is a multi-organ transplant recipient
2. Male or female subject ≥ 65 years old	2. Recipient of bloodgroup ABO incompatible allograft or CDC cross-match positive transplant
3. Subject randomized within 24 h of completion of transplant surgery	3. Subject at high immunological risk for rejection as determined by local practice for assessment of anti-donor reactivity
4. Stratum A: Recipient of a primary (or secondary, if first graft is not lost due to immunological reasons) renal transplant from a deceased donor aged 65 years or older	4. Recipient of a kidney with a cold ischemia time (CIT) > 24 h
5. Stratum B: Recipient of a primary (or secondary, if first graft is not lost due to immunological reasons) renal transplant from a deceased donor aged below 65 years or a living donor of any age	5. Recipients of a kidney from an HLA-identical related living donor
	6. Known intolerability for one or more of the study drugs
	7. Subject who is HIV positive
	8. HBsAg and/or a HCV positive subject with evidence of elevated liver function tests (ALT/AST levels ≥ 2.5 times ULN). Viral serology results obtained within 6 months prior to randomization are acceptable
	9. Recipient of a kidney from a donor who tests positive for human immunodeficiency virus (HIV), hepatitis B surface antigen (HBsAg) or anti-hepatitis C virus (HCV)
	10. Subject with severe systemic infections, current or within the two weeks prior to randomization
	11. Subject with severe restrictive or obstructive pulmonary disorders
	12. Subject with severe hypercholesterolemia or hypertriglyceridemia that cannot be controlled
	13. Subject with white blood cell (WBC) count ≤ 2,000/mm^3^ or with platelet count ≤ 50,000/mm^3^

### Randomization and study treatment

Patients will be randomized at transplantation to receive either a standard quadruple immunosuppression regimen with tacrolimus and mycophenolate mofetil (the ‘Tacrolimus, mycophenolate mofetil, prednisolone group’ (TMP group)), or a low exposure tacrolimus regimen in combination with everolimus (the ‘Tacrolimus, everolimus, prednisolone group’ (TEP-group). Randomization is based on the stratum.

Patients will be randomized for the TMP group or the TEP group in a 1:1 ratio. They will be randomized within 24 h of completion of the transplant surgery. The randomization procedure is done through a web-based system (ALEA) (https://www.aleaclinical.eu/). Patients are randomized with block randomization. In stratum A, patients are stratified per center, and in stratum B patients are stratified per center for both living donors and deceased donors.

**Fig. 1:**
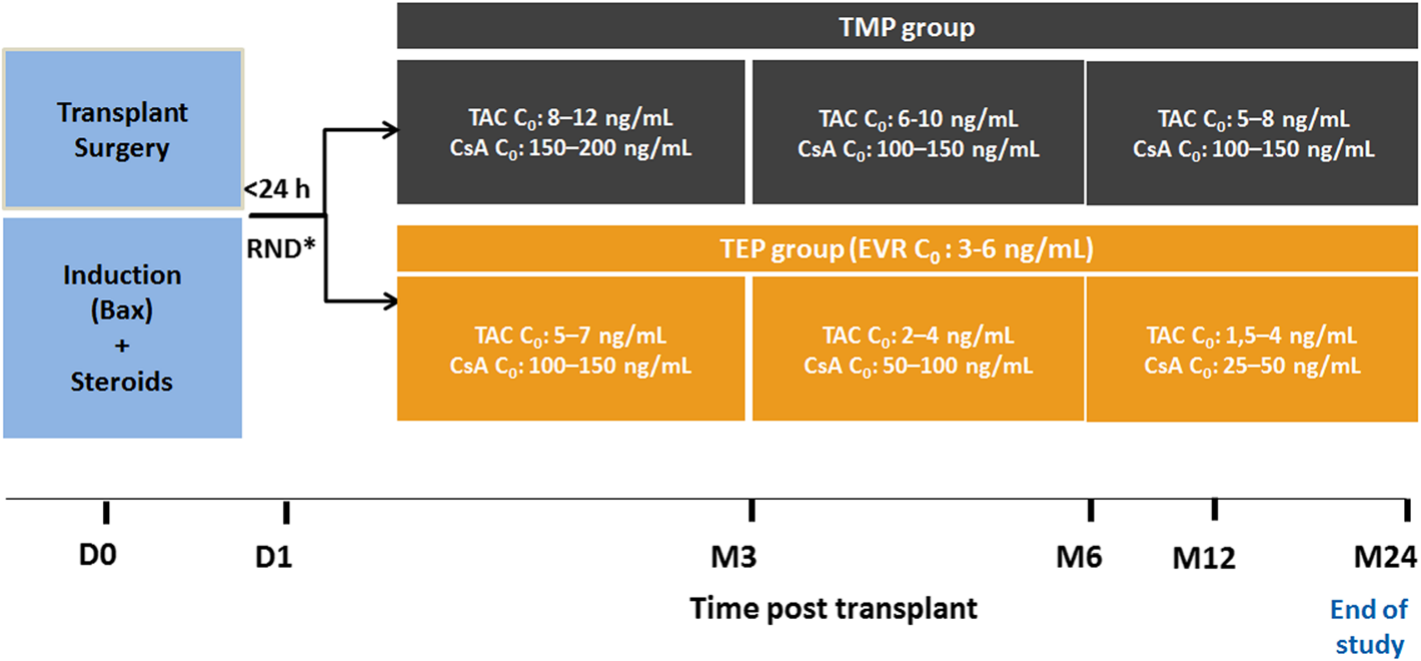
OPTIMIZE study design. TMP = tacrolimus, mycophenolate mofetil, prednisolon, TAC = tacrolimus, CsA = cyclosporine A, BAX = basiliximab, RND = randomization, TEP = tacrolimus, everolimus, prednisolone, EVR = everolimus

Two immunosuppressive regimens will be used in this study (see Fig. 1).

All patients will receive intravenous induction therapy with two doses of basiliximab; 20 mg on day 0 and 20 mg on day 4. Alternative induction therapy with T cell-depleting agents is not permitted.

All patients will start with glucocorticoids on day 0, which is tapered to a daily dose of (an equivalent dose of) 5 mg prednisolone after 3 months.

Treatment will be started with tacrolimus once-daily (Envarsus® (Chiesi Pharmaceuticals B.V.) or Advagraf® (Astellas Pharma), if Envarsus ® is not available. A twice-daily tacrolimus formulation can be used if deemed necessary by the local investigator. If tacrolimus is not tolerated, it may be replaced by cyclosporine.

Interruptions of everolimus, mycophenolate mofetil, or CNI are allowed for 21 consecutive days within the study protocol. In elective cases, everolimus might be interrupted an extra 5 consecutive days before any surgery.

Patients that are randomized to the TMP group, will receive a starting dose of 7 mg Envarsus® *qd* (or an equivalent dose of Advagraf®), with an initial target trough concentration of 8–12 ng/ml tapered to 6–10 from 3 months onward, and 5–8 ng/ml from 6 months after transplantation. Mycophenolate mofetil will be given in a dose of 500 mg bid throughout the trial, and will not be adapted to trough concentrations nor AUC.

Patients that are randomized to the TEP group, will receive a starting dose of 5 mg Envarsus® *qd* (or an equivalent dose of Advagraf®) qd with an initial target trough concentration of 5–7 tapered to 2–4 ng/ml from 3 months onwards, and 1.5-4 ng/ml from 6 months after transplantation. Everolimus will be initiated at a starting dose of 1.5 mg *bid* with a target trough concentration of 3–6 ng/ml throughout the trial.

#### Concomitant medication

##### Cytomegalovirus prophylaxis

Cytomegalovirus (CMV) prophylaxis is recommended for all donor CMV positive/recipient CMV negative cases and considered for all recipients who are CMV positive. It is recommended that CMV prophylaxis be administered for a minimum of three months after transplantation.

##### Pneumocystis jirovecii (Pneumocystis carinii) pneumonia prophylaxis

All subjects will be started on trimethoprim-sulfamethoxazole when oral medication can be tolerated, and continued until at least six months post-transplant. After six months, subjects will be treated per local practice. Aerosolized pentamidine, dapsone or atovaquone may be administered to subjects who are unable to tolerate trimethoprim-sulfamethoxazole.

##### Hyperlipidemia

Lipid lowering medications should be administered according to guidelines and local practice.

##### Hepatitis B (HBV) prophylaxis

Prophylaxis for recurrent hepatitis B during the course of this study is allowed and will be administered at the discretion of the investigator.

##### Prohibited treatment

Treatment with sirolimus, belatacept and azathioprine is not allowed.

### Outcomes

#### The primary endpoint

This study will evaluate two immunosuppressive regimens in elderly KTR in two strata with adapted endpoints based on the expected outcome according to donor characteristics. Both strata will be analyzed together for the primary endpoint.

The primary endpoint will be “successful transplantation”, which is defined as survival with a functioning allograft with an estimated GFR (eGFR) above 30 (stratum A) or 45 (stratum B) ml/min per 1.73 m^2^ at two years after transplantation. We chose to stratify the endpoint for donor type as a substantial proportion of kidney function after transplantation is determined by donor type, and we.

only expect medication-related modification of kidney function within this donor-related predetermined potential of the transplanted kidney.

#### Secondary endpoints

The main secondary endpoint is the primary endpoint analyzed separately per stratum.

Other secondary objectives include assessment at months 12 and 24 of death, graft loss and eGFR below 30 or 45 ml/min/1.73m^2^.

Secondary endpoints include the incidence of (biopsy-proven) rejection, presence of and changes in immunosenescence, frailty and co-morbidities, and assessment of and changes in health-related quality of life as a patient-reported outcome. All endpoints will be analyzed in both the complete study population and the separate strata.

Safety objectives include standard assessments of (serious) adverse events (including drug-related adverse events and drug-related discontinuation of study medication), and specific objectives regarding clinically relevant infections, post-transplantation diabetes mellitus, malignancy and cardiovascular events. In Table 2 a complete overview of the study endpoints is depicted. Finally, a cost-effectiveness analysis will also be performed.

To assess immunosenescence we will assess the immunological phenotype pre-transplant and identify T cell ageing characteristics that are associated with the risk for acute rejection and infection after kidney transplantation. We also will assess immunological ageing within a two year period after kidney transplantation in relation to the two different immunosuppressive regimens.

See Table 2 for a complete overview of the endpoints.

**Table 2:** Primary and secondary endpoints of the OPTIMIZE study

	Endpoint	Determination of endpoint
**Primary endpoint**	‘successful transplantation’	At 24 months after transplantation: survival with a functioning allograft with an estimated GFR above 30 (stratum A) or 45 (stratum B) ml/min per 1.73 m^2^.
**Main secondary endpoint**	Primary objective, analyzed separately per stratum	-
**Other secondary endpoints**	Incidence of individual endpoints of death, graft loss, eGFR below 30 or 45 ml/min/1.73m^2^	At 12 and 24 months after transplantation
	Evolution of renal function over time by slope analysis	eGFR and creatinine clearance, based on the 24 hour excretion of creatinine in the urine
	Incidence of treated biopsy-proven rejection (tBPAR)	Will be checked and recorded at every study visit
	Rejection treatment and type of rejection treatment	Will be checked and recorded at every study visit
	Development of donor-specific anti-HLA antibodies	At 12 and 24 months after transplantation: DSA as measured by Luminex
	Incidence of (drug-related)adverse events, serious adverse events and adverse reactions, including drug-related discontinuation of study medication	All adverse events reported spontaneously by the subject or observed by the investigator or his staff will be recorded
	Incidence of clinically relevant infections, post transplantation diabetes mellitus, malignancies and cardiovascular events	All adverse events reported spontaneously by the subject or observed by the investigator or his staff will be recorded
	Presence of frailty after transplantation and change in frailty from baseline	At baseline: clinical frailty score and hand grip strength. At 12 and 24 months after transplantation: clinical frailty score, hand grip strength, Fried Frailty Index
	Physical and cognitive functioning and changes over time	At 12 and 24 months after transplantation: Short Physical Performance Battery, Montreal Cognitive Assessment
	Health-related quality of life at 0, 12 and 24 months and changes from baseline	Questionnaires: Short-Form-12 and European Quality of life-5 Dimensions
	Difference in illness perception at 0, 12 and 24 months and changes from baseline	Questionnaire: Brief Illness Perception Questionnaire
	Difference in symptoms at 0, 12 and 24 months and changes from baseline	Questionnaire: Dialysis Symptom Index with additional items from the Modified Transplant Symptom Occurrence and Symptom Distress Scale-59
	Difference in adherence of immunosuppressive medication at 12 and 24 months	Questionnaire: Basel Assessment of Adherence to Immunosuppressive Medication Scale
	Presence of markers for immunosenescence at 12 and 24 months and changes from baseline	T cell differentiation, exhaustion and telomere length will be assessed by flowcytometry of Peripheral Blood Mononuclear Cells
	Difference in iBOX predicted outcome at 3, 5 and 7 years	Based on the available data
	Development of a pharmacokinetic model for tacrolimus once-daily (Envarsus®), using data on AUC’s	In addition to trough levels, additional AUC’s will be withdrawn at the Leiden University Medical Center as routine patient care on week 2 and 6.
**Other**	Evaluation of Cost-effectiveness of the new immunosuppressive regimen, and comparison to the current standard of care	Cost-effectiveness of the immunosuppressive regimen will be evaluated using state-of-the-art health-economic techniques; costs and effectiveness of immunosuppressive therapy will be derived from the study

### Study procedures and data management

In this study, there will be 9 study visits: at baseline, 7 days, 4 weeks, 3, 6, 9, 12, 18 and 24 months after transplantation. For most visits, only vital signs, blood- and urine analysis will be done. At some visits, extra tests and/or questionnaires will be done. See Table 3 for a complete overview.

**Table 3:** Timing of study procedures

	BASELINE	Day 7	Week 4	Mo 3	Mo 6	Mo 9	Mo 12	Mo 18	Mo 24
Day	0	7	28	90	180	270	360	540	720
**Time window (+/- days)**		+/-2	+/-7	+/-14	+/-21	+/-21	+/-21	+/-21	+/-21
**Demographics**	X								
**Medical history**	X								
**Donor & transplantation related information**	X								
**Dialysis information**	X	X	X	X					
**Vital signs -** *Height (only at baseline), blood pressure, weight, heart rate*	X	X	X	X	X	X	X	X	X
**Trough levels CNI/EVR**		X	X	X	X	X	X	X	X
**Haematology -** *Hb, Ht, MCV, leucocytes + differentiation, platelets*	X		X	X	X	X	X	X	X
**Biochemistry -** *Creatinine, sodium, potassium, albumin, calcium, phosphate, glucose, HbA1c*	X		X	X	X	X	X	X	X
**Lipid Profile -** *Cholesterol (total, HDL and LDL) and triglycerides*	X			X	X	X	X	X	X
**24 hour Urine -** *Protein, sodium, creatinine clearance*					X		X		X
**Spot urine -** *Creatinine, protein*	X		X	X	X	X	X	X	X
**Virology** ***–*** *Cytomegalovirus PCR, BK-virus PCR*				X	X		X		
**Biobanking** – *10 ml EDTA, 10 ml serum, 8 ml urine (urine not at baseline)*	X				X		X		X
**Optional: PBMCs –** *3 x 10 ml lithium heparin (+ lymphocyte subset)*	X						X		X
**DSA**	X						X		X
**Tests and questionnaires 1** *- Grip strength, CFS, SF-12, EQ-5D, B-IPQ, DSI + MTSOSD-59*	X						X		X
**Tests and questionnaires 2 –** FFI, SPPB, MoCA, BAASIS							X		X
**Secondary endpoint status**		X	X	X	X	X	X	X	X
**Study medication status**		X	X	X	X	X	X	X	X
**Concomitant medication**	X	X	X	X	X	X	X	X	X
**Adverse events**	X	X	X	X	X	X	X	X	X

*PBMCs* Peripheral Blood Mononuclear Cells, *DSA* donor-specific anti-HLA antibodies, *CFS* Clinical Frailty Scale, *SF-12* Short Form -12 (SF-12), *EQ-5D* European Quality of life-5 Dimensions, *B-IPQ* Brief Illness Perception Questionnaire, *DSI* Dialysis Symptom Index, *MTSOSD-59* Modified Transplant Symptom Occurrence and Symptom Distress Scale-59, *FFI* Fried Frailty Index, *MoCA* Montreal Cognitive Assessment, *SPPB* short physical performance battery, *BAASIS* Basel Assessment of Adherence to Immunosuppressive Medication Scale

All procedures will follow standard procedures, except biobanking. For this purpose, extra blood samples will be withdrawn, at baseline, and 6, 12 and 24 months after transplantation. These will be stored in a biobank. A special part of the biobank is the collection of isolated Peripheral Blood Mononuclear Cells (PBMCs); this will we done for 250 patients. The PBMCs will be used for study of immunosenescence.

Several instruments, both objective and subjective, will be used to measure frailty, cognitive and physical functioning, health-related quality of life, illness perceptions, symptom burden and adherence to immunosuppressive medication of the KTR’s in this study. See Additional file 1 I for more information regarding tests and questionnaires.

Loss to follow-up virtually does not exist in the transplant population. If patients withdraw their informed consent, we will ask their permission to obtain a creatinine value and dialyses information at months 6, 12 and 24. Patients that discontinue their randomized study regimen (for more than 21 days) will remain in the study.

All study data will be entered into the secured OPTIMIZE project database. This database complies with the standards of Good Clinical Practice.

### Safety and monitoring

The assessment of safety will be based mainly on the frequency of adverse events, which includes all serious adverse events. Adverse events will be summarized by presenting the number and percentage of patients having any adverse event. Any other information collected (e.g. severity or relatedness to study medication) will be listed as appropriate. An independent data safety monitoring board will regularly review the safety data, efficacy data and the events of interest. Monitoring will be executed in compliance with the guideline “Quality Assurance of research involving human subjects 2.0” of “The Netherlands Federation of University Medical Centers” [22]. Intensive monitoring for this study will be performed by an independent and qualified monitor of the Service Desk Clinical Research Office of the University Medical Centre Groningen.

### Sample size

The cut-off values for the primary endpoint is based on a previous analysis of results of kidney transplantation in the elderly (≥ 65 years) in The Netherlands [1]. This analysis demonstrated that about 50 % of elderly patients who received a kidney from an older (≥ 65 years) donor have an eGFR above 30 ml/min/1.73m^2^ at one year after transplantation. For elderly recipients of a kidney from a donor younger than 65 years about 50 % have an eGFR above 45 ml/min/1.73m^2^ at one year after transplantation. The latter results resemble the expected results of living donor transplantation in this age group where the majority of donors are ≥ 65 years.

Based on the above, we expect a successful transplantation rate of 48 % of the KTR in the TMP group at two years after transplantation. For the TEP group, a 14 % higher success rate of 62 % is expected.

A total of 374 patients will be included in the study: 97 patients per arm in stratum A, and 90 patients per arm in stratum B. Two groups of *n *= 187 will, using a one-sided T-test, lead to a power of at least 0.86 of finding a 14 % difference between the two groups for the primary endpoint.

### Statistical analysis

The data from all centers will be pooled and summarized with respect to demographic and baseline characteristics and efficacy and safety observations. Exploratory analyses will be performed using descriptive statistics. The primary outcome variable ‘successful transplantation’ will be analyzed using a generalized mixed model, adjusting for stratum (A/B) and center. Data will be presented for the complete intention-to-treat population (all patients having taken at least one dose of study medication), as well as the per-protocol population (all patients who completed the study without major protocol deviations). For missing data we will perform regression based multiple imputation.

### Trial status

The trial started in July 2019. On 01-04-2021, all centers had started and a total of 116 patients were included: 64 in stratum A and 52 in stratum B. A total inclusion time of 4 years was foreseen, based on the number of transplantations in the years 2014–2016, and the consent rate of other trials. However, due to the COVID-19 pandemic study inclusion will be delayed, although the extent is unclear yet.

## Discussion

Here we describe the design of the OPTIMIZE study; a trial that compares two immunosuppressive regimens in 374 *de novo*, elderly KTR.

Elderly patients make up one third of the total population of KTR in The Netherlands and have different characteristics when compared to younger recipients. Elderly recipients experience less rejection related graft loss, but more immunosuppression-related graft loss. We felt that it was of utmost importance to conduct a randomized clinical trial that may lead to optimization of the immunosuppressive treatment – and thereby, better outcomes - for this large, and growing, distinct population.

Our study design resembles that of the TRANSFORM study. As described above, in this study no difference in kidney transplant function was observed at two years after transplantation. To enhance the chance of reaching a difference in the primary outcome between the two groups, we aim for lower tacrolimus trough concentrations in the TEP (= reduced CNI) group than was intended in the TRANSFORM study. We aim for levels of 5–7 ng/ml during months 0–3, 2–4 ng/ml (instead of 2–5) during months 3–6, and 1.5–4 ng/ml (instead of 2–4) thereafter. We will closely review the tacrolimus trough concentrations for all patients on a monthly basis to ensure that target levels are achieved in our study.

Our primary endpoint is ‘successful transplantation’. We did not include tBPAR in the primary endpoint. Several studies have already shown that the risk for acute rejection in the elderly is low, especially when the kidney is from a younger donor (< 65 years) or from an elderly (≥ 65 years) donation after brain death donor [1, 23]. Because we examine a low to medium risk population, we expect the acute rejection risk to be even lower in our elderly population. Additionally, the TRANSFORM study already showed that rates of tBPAR did not differ between the reduced- and standard CNI group. tBPAR was therefore included as a secondary endpoint only.

One of the ‘standard’ secondary endpoints is the evolution of renal function over time by slope analysis. We will use both eGFR and creatinine clearance, based on the 24-hour urinary excretion of creatinine. A recent study showed that, surprisingly enough, inhibition of mTORC1 in sarcopenic rats counteracted sarcopenia, as determined by observing an increase in muscle mass [24]. Although this has not been studies in humans, we hypothesize that everolimus may lead to an increase in muscle mass, which can be reflected by a rise in the serum creatinine and a drop in the eGFR. This and the frequently observed increase in muscle mass in transplant recipients is why we feel that using the eGFR alone for assessment of evolution of kidney function over time might not be sufficient.

In addition to some ‘standard’ secondary endpoints, we will pay special attention to frailty and health-related quality of life of the elderly KTR. This will increase our knowledge on the benefits and risks of transplantation in elderly patients and will better fit the outcomes of interest for older patients. We will use the trial data to assess the cost-effectiveness of the TEP-regimen, compared to the TMP-regimen.

This study will also provide information on the function of the aged immune system after kidney transplantation and the associated effect of the two different drug regimens. We aim to identify immunologic parameters that may serve as biomarkers of immunosenescence for future clinical application.

We want to mention a few limitations of our study. The first one is that the trial is not powered for differences of smaller than 14 % between the two groups for the primary endpoint ‘successful transplantation’. However, this was a deliberate choice; the study group decided that differences below 14 % would probably not be clinically relevant. The second one is that the study is not blinded. As we need to adjust the dose of the study medication according to the trough concentrations, unfortunately, blinding is nearly impossible. A third limitation is the fact that induction with T cell-depleting therapy is not allowed. This is because induction with T cell-depleting therapy is not part of standard practice in this vulnerable patient group in the Netherlands and Belgium.

## Conclusions

The OPTIMIZE study is a unique clinical trial; it is the first randomized clinical trial to extensively examine the effect of a low exposure tacrolimus regimen in combination with everolimus specifically in *de novo* elderly kidney transplant recipients. The study also pays attention to the quality of life, cognitive and physical functioning of the participants. The unique character of the study in combination with the data it will yield will position the OPTIMIZE study to have a profound impact on future kidney transplant practice in elderly recipients.

## Supplementary Information

**Additional file 1.**

## Data Availability

The datasets used and/or analysed during the current study are available from the corresponding author on reasonable request.

## Abbreviations

KTR: Kidney transplant recipients
ESP: Eurotransplant Senior Program
CNIs: Calcineurin inhibitors
HIV: Human immunodeficiency virus
HBsAg: Hepatitis B surface antigen
HVC: Anti-hepatitis C virus (HCV)
TMP group: Tacrolimus, mycophenolate mofetil, prednisolone group
TEP-group: Tacrolimus, everolimus, prednisolone group
TAC: Tacrolimus
CsA: Cyclosporine A
BAX: Basiliximab
RND: Randomization
EVR: Everolimus
HBV: Hepatitis B
eGFR: Estimated GFR
tBPAR: Treated biopsy-proven rejection
PBMCs: Peripheral Blood Mononuclear Cells
DSA: Donor-specific anti-HLA antibodies
CFS: Clinical Frailty Scale
*SF-12*: Short Form − 12
EQ-5D: European Quality of life-5 Dimensions
B-IPQ: Brief Illness Perception Questionnaire
DSI: Dialysis Symptom Index
MTSOSD-59: Modified Transplant Symptom Occurrence and Symptom Distress Scale-59
FFI: Fried Frailty Index
MoCA: Montreal Cognitive Assessment
SPPB: Short physical performance battery
BAASIS: Basel Assessment of Adherence to Immunosuppressive Medication Scale
