# Supplementary material for: Rationale and design of the OPTIMIZE trial: OPen label multicenter randomized trial comparing standard IMmunosuppression with tacrolimus and mycophenolate mofetil with a low exposure tacrolimus regimen In combination with everolimus in de novo renal transplantation in Elderly patients
Source: BMC Nephrol. 2021 Jun 2;22:208. doi: 10.1186/s12882-021-02409-8 (PMC8172178; doi:10.1186/s12882-021-02409-8)
Supplement: Supplementary file 1 — Additional file 1. [file 12882_2021_2409_MOESM1_ESM.docx]

**Supplemental Appendix 1 – Background information about tests and questionnaires used in the OPTIMIZE study**

**Frailty**  Frailty is assessed by two measures: The Clinical Frailty Scale (CFS) and the Fried Frailty Index (FFI). Both measures are validated instruments to establish prevalence rates for frailty.

The CFS is a subjective measure of frailty based on clinical judgements and uses clinical descriptors and pictographs to assess the level of vulnerability from 1 (very fit) to 9 (terminally ill). The CFS has shown to have a high inter-rater reliability (ICC 0.97) and to correlate well with objective measures of frailty (r = 0.80).[1] For subgroup analyses, CFS scores can be subdivided into three categories according to functional ability: managing well (CFS score 1-3); vulnerable to mildly frail (CFS score 4-5); and moderate to severe frail (CFS score 6-9). Patients classified into the latter two categories will be considered as frail.

The FFI is an objective, validated measure of frailty, consisting of five criteria: weakness, slow walking speed, low physical activity, self-reported exhaustion, and unintentional weight loss.[2] Each item of the FFI is awarded a score of 1 if present. A summary score is indicative of: non-frail (score 0), pre-frail (score 1-2) or frail (score 3-5). Patients classified into the latter two categories will be considered as frail.

**Physical functioning**  To measure physical functioning the Dutch version of the short physical performance battery (SPPB) is used. The SPPB is a group of measures that combines the results of the gait speed, chair stand and balance tests. It has been used as a predictive tool for possible disability and can aid in the monitoring of function in older people. The scores range from 0 (worst performance) to 12 (best performance). The SPPB has been shown to have good properties regarding validity, reliability, and responsiveness.[3]

**Cognitive functioning**  Because frailty in elderly is often combined with a decline in cognitive functioning, the cognitive functioning will also be assessed using the Dutch version of the Montreal Cognitive Assessment (MoCA).[4, 5] This 10-minute assessment covers visio-spatial and executive functioning, naming, memory, attention, language, abstraction, delayed recall and orientation. The MoCA’s sensitivity and specificity exceed those of the Mini Mental State Examination, regarding detection of mild cognitive impairment in older community-dwelling adults and haemodialysis patients.[6, 7] Possible scores range from 0-30; scores <26 indicate cognitive impairments.

**Health related quality of life**  Two instruments will be used to measure quality of life, the Short Form -12 (SF-12), and the European Quality of life-5 Dimensions (EQ-5D). The SF-12 is the short-form of the SF-36 health survey, a widely used and well validated health-related quality-of-life questionnaire. The SF-12 consists of twelve questions that measure eight health domains to assess physical and mental health. The SF-12 summary scores for physical functioning (PCS-12) and mental functioning (MCS-12) range from 0 to 100, with higher scores representing better self-reported health. The instrument has been validated across a number of chronic diseases and conditions including kidney disease.[8]

The EQ-5D is a standardised measure of health status in order to provide a simple, generic measure of health for clinical and economic appraisal. The EQ-5D consists of 5 dimensions (mobility, self-care, usual activities, pain/discomfort, and anxiety/depression) and a numerical expression of self-perceived health status. Each dimension has three possible answers: no problems, some problems, and extreme problems. The EQ VAS records the respondent’s self-rated health on a visual analogue scale with endpoints labelled ‘the best health you can imagine’ and ‘the worst health you can imagine’. This information can be used as a quantitative measure of health as judged by the individual respondents. The EQ5D was found to be a valid instrument for the measurement of health status in renal transplant patients, with good construct, concurrent and discriminative validity.[9]

*Illness perceptions –* A growing body of literature suggests that patients’ illness perceptions are key to understanding why many patients with chronic kidney disease have poor outcomes that are not explained by the clinical severity of the disease, including an impaired health-related quality of life, a faster disease progression, depressive symptoms and mortality.[10] Moreover, these illness perceptions seem to be a promising starting point for patient-centred interventions to improve clinical and patient-reported outcomes. The commonly used and validated Brief Illness Perception Questionnaire[11] (B-IPQ) will be used to measure patients’ beliefs about their illness and treatment. The B-IPQ assesses the following eight illness perceptions by means of a single item using a 0-to-10 response scale: illness identity, timeline acute/chronic, negative consequences, personal control, treatment control, illness coherence, emotional response and illness concern.

**Symptom burden-** It is increasingly recognized that patient-reported outcomes such as symptom experience are crucial for understanding the benefit and/or burden of treatment, for understanding patients’ health-related quality of life and (non)adherence, and to detect issues that might jeopardize patients’ clinical outcome. An adjusted questionnaire consisting of the complete Dialysis Symptom Index (DSI) with additional items from the Modified Transplant Symptom Occurrence and Symptom Distress Scale-59 Items (MTSOSD-59) is developed in collaboration with nephrologists and patients to capture the vast range of symptoms experienced by kidney transplant recipients.

The DSI is a 30-item disease-specific symptom questionnaire to assess physical and emotional symptom burden. A recent study found the DSI to be valid and reliable, the most relevant, complete, and comprehensible symptom questionnaire available for routine assessment in patients with CKD, including kidney transplant recipients.[12] In total, 32 items of the 59 MTSOSD items were added to assess symptoms associated with side effects of immunosuppressive medication. The adjusted questionnaire consists of 62 items, with each item reflecting one symptom that is scored for symptom occurrence and symptom burden. Symptom occurrence is assessed with the question “During the past week: did you experience this symptom?” using a yes or no format. Symptom burden is measured using the question “If yes, how much did it bother you?” using a 5-point rating scale ranging from 0 (not at all) to 4 (very much). A total sum score of symptom occurrence and symptom burden is calculated, with higher scores indicating higher symptom burden. To ensure comprehensiveness for individual patients, an open-ended question is added to report three additional symptoms.

**Adherence to immunosuppressive medication** The magnitude of nonadherence to immunosuppressive medication in kidney transplant recipients is high and nonadherence is associated with adverse outcomes such as graft loss. In this study, adherence to immunosuppressive medication is assessed by means of the Basel Assessment of Adherence to Immunosuppressive Medication Scale (BAASIS).[13] The questionnaire consists of four items referring to the four dimensions of medication adherence, namely: 'Taking dimension’, 'Drug holidays', 'Timing dimension' and 'Reduction of dose of medication'. For each domain, occurrence of nonadherence during the last 4 weeks is assessed using a yes or no format, followed by the assessment of nonadherence frequency using a 6-point scale (i.e. the answer categories: Never, Once a month, Every two weeks, Every week, More than once a week, and Every day).

**References:**

1. Rockwood K, Song X, MacKnight C, Bergman H, Hogan DB, McDowell I, et al. A global clinical measure of fitness and frailty in elderly people. CMAJ. 2005;173:489–95.

2. Fried LP, Tangen CM, Walston J, Newman AB, Hirsch C, Gottdiener J, et al. Frailty in Older Adults: Evidence for a Phenotype. Journals Gerontol Ser A Biol Sci Med Sci. 2001;56:M146–57.

3. Freiberger E, De vreede P, Schoene D, Rydwik E, Mueller V, Frändin K, et al. Performance-based physical function in older community-dwelling persons: A systematic review of instruments. Age Ageing. 2012;41:712–21.

4. Nasreddine ZS, Phillips NA, BÃ©dirian V, Charbonneau S, Whitehead V, Collin I, et al. The Montreal Cognitive Assessment, MoCA: A Brief Screening Tool For Mild Cognitive Impairment. J Am Geriatr Soc. 2005;53:695–9.

5. Thissen AJAM, Van Bergen F, De Jonghe JFM, Kessels RPC, Dautzenberg PLJ. Bruikbaarheid en validiteit van de Nederlandse versie van de montreal cognitive assessment (MoCA-D) bij het diagnosticeren van mild cognitive impairment. Tijdschr Gerontol Geriatr. 2010;41:231–40.

6. Trzepacz PT, Hochstetler H, Wang S, Walker B, Saykin AJ. Relationship between the Montreal Cognitive Assessment and Mini-mental State Examination for assessment of mild cognitive impairment in older adults. BMC Geriatr. 2015;15:107.

7. Tiffin-Richards FE, Costa AS, Holschbach B, Frank RD, Vassiliadou A, Krüger T, et al. The Montreal Cognitive Assessment (MoCA) - A sensitive screening instrument for detecting cognitive impairment in chronic hemodialysis patients. PLoS One. 2014;9.

8. Lacson E, Xu J, Lin SF, Dean SG, Lazarus JM, Hakim RM. A comparison of SF-36 and SF-12 composite scores and subsequent hospitalization and mortality risks in long-term dialysis patients. Clin J Am Soc Nephrol. 2010;5:252–60.

9. Cleemput I, Kesteloot K, Moons P, Vanrenterghem Y, Van Hooff JP, Squifflet JP, et al. The construct and concurrent validity of the EQ-5D in a renal transplant population. Value Heal. 2004;7:499–509.

10. Clarke AL, Yates T, Smith AC, Chilcot J. Patient’s perceptions of chronic kidney disease and their association with psychosocial and clinical outcomes: A narrative review. Clinical Kidney Journal. 2016;9:494–502.

11. Broadbent E, Wilkes C, Koschwanez H, Weinman J, Norton S, Petrie KJ. A systematic review and meta-analysis of the Brief Illness Perception Questionnaire. Psychol Heal. 2015;30:1361–85.

12. Van Der Willik EM, Meuleman Y, Prantl K, Van Rijn G, Bos WJW, Van Ittersum FJ, et al. Patient-reported outcome measures: Selection of a valid questionnaire for routine symptom assessment in patients with advanced chronic kidney disease - A four-phase mixed methods study. BMC Nephrol. 2019;20:344.

13. Dobbels F, Berben L, De Geest S, Drent G, Lennerling A, Whittaker C, et al. The psychometric properties and practicability of self-report instruments to identify medication nonadherence in adult transplant patients: A systematic review. Transplantation. 2010;90:205–19.
